# A multicenter cohort study on the association between prehospital immobilization and functional outcome of patients following spinal injury in Asia

**DOI:** 10.1038/s41598-022-07481-0

**Published:** 2022-03-03

**Authors:** Hsuan An Chen, Shuo Ting Hsu, Sang Do Shin, Sabariah Faizah Jamaluddin, Do Ngoc Son, Ki Jeong Hong, Hideharu Tanaka, Jen Tang Sun, Wen Chu Chiang, The PATOS Clinical Research Network, T. V. Ramakrishnan, Sabariah Faizah Jamaluddin, Hideharu Tanaka, Bernadett Velasco, Ki Jeong Hong, Jen Tang Sun, Pairoj Khruekarnchana, Saleh Fares LLC, Do Ngoc Son, Ramana Rao, George P. Abraham, T. V. Ramakrishnan, Sabariah Faizah Jamaluddin, Mohd Amin Bin Mohidin, Al-Hilmi Saim, Lim Chee Kean, Cecilia Anthonysamy, Shah Jahan Din Mohd Yssof, Kang Wen Ji, Cheah Phee Kheng, Shamila bt Mohamad Ali, Periyanayaki Ramanathan, Chia Boon Yang, Hon Woei Chia, Hafidahwati Binti Hamad, Samsu Ambia Ismail, Wan Rasydan B. Wan Abdullah, Hideharu Tanaka, Akio Kimura, Bernadett Velasco, Carlos D. Gundran, Pauline Convocar, Nerissa G. Sabarre, Patrick Joseph Tiglao, Ki Jeong Hong, Kyoung Jun Song, Joo Jeong, Sung Woo Moon, Joo-yeong Kim, Won Chul Cha, Seung Chul Lee, Jae Yun Ahn, Kang Hyeon Lee, Seok Ran Yeom, Hyeon Ho Ryu, Su Jin Kim, Sang Chul Kim, Ray-Heng Hu, Jen Tang Sun, Ruei-Fang Wang, Shang-Lin Hsieh, Wei-Fong Kao, Sattha Riyapan, Parinya Tianwibool, Phudit Buaprasert, Osaree Akaraborworn, Omer Ahmed Al Sakaf, Saleh Fares LLC, Le Bao Huy, Do Ngoc Son, Nguyen Van Dai

**Affiliations:** 1 Department of Emergency Medicine, Far Eastern Memorial Hospital, New Taipei City, Taiwan; 2 Department of Emergency Medicine, Seoul National University College of Medicine and Hospital, Seoul, Korea; 3 Faculty of Medicine, Universiti Teknologi MARA, Shah Alam, Malaysia; 4 Center for Critical Care Medicine, Bach Mai Hospital, Hanoi, Vietnam; 5 Department of Emergency and Critical Care Medicine, Hanoi Medical University, Hanoi, Vietnam; 6 Faculty of Medicine, University of Medicine and Pharmacy, Vietnam National University, Hanoi, Vietnam; 7 Department of Emergency Medical System, Graduate School of Kokushikan University, Tokyo, Japan; 8 School of Medicine, Tzu Chi University, Hualien City, Taiwan; 9 Department of Emergency Medicine, National Taiwan University Hospital, Taipei, Taiwan; 10 Department of Emergency Medicine, National Taiwan University Hospital, Yun-Lin Branch, Taipei, Taiwan; 11 Sri Ramachandra Medical College, Chennai, India; 12 East Avenue Medical Center, Quezon City, Philippines; 13 Ravajith Hospital, Bangkok, Thailand; 14 National Ambulance, Abu Dhabi, United Arab Emirates; 15 GVK EMRI, Hyderabad, India; 16 Indian Institute of Emergency Medical Services, Chennai, India; 17 Sultanah Aminah Hospital, Johor, Malaysia; 18 Seri Manjung Hospital, Seri Manjung, Malaysia; 19 Pulau Pinang Hospital, Pulau Pinang, Malaysia; 20 Serdang Hospital, Kajang, Malaysia; 21 Kuala Lumpur Hospital, Kuala Lumpur, Malaysia; 22 Sarikei Hospital, Sarikei, Malaysia; 23 Sabah Women and Childrens Hospital, Kota Kinabalu, Malaysia; 24 Ampang Hospital, Ampang, Malaysia; 25 Kajang Hospital, Kajang, Malaysia; 26 Miri Hospital, Miri, Malaysia; 27 Sarawak General Hospital, Kuching, Malaysia; 28 Queen Elizabeth II Hospital, Kota Kinabalu, Malaysia; 29 Teluk Intan Hospital, Teluk Intan, Malaysia; 30 Raja Perempuan Zainab II Hospital, Kota Bharu, Malaysia; 31 National Center for Global Health and Medicine Hospital, Tokyo, Japan; 32 Philippine College of Emergency Medicine, Parañaque, Philippines; 33 Southern Philippines Medical Centre, Davao, Philippines; 34 Pasig City General Hospital, Pasig, Philippines; 35 Corazon Locsin Montelibano Memorial Regional Hospital, Bacolod, Philippines; 36 Seoul National University Hospital, Seoul, South Korea; 37 Boramae Medical Center, Seoul, South Korea; 38 Seoul National Univerisity Bundang Hospital, Seoul, South Korea; 39 Korea University Ansan Hospital, Ansan, South Korea; 40 Samsung Medical Center, Seoul, South Korea; 41 Dongguk University Ilsan Hospital, Goyang, South Korea; 42 Kyungpook National University Hospital, Daegu, South Korea; 43 Wonju Severance Christian Hospital, Wonju, South Korea; 44 Pusan National University Hospital, Busan, South Korea; 45 Chonnam National University Hospital, Gwangju, South Korea; 46 Korea University Anam Hospital, Seoul, South Korea; 47 Chungbuk National University Hospital, Cheongju, South Korea; 48 National Taiwan University Hospital, Taipei, Taiwan; 49 Shin Kong Wu Ho-Su Memorial Hospital, Taipei, Taiwan; 50 Mackay Memorial Hospital, Taipei, Taiwan; 51 Taipei City Hospital, Taipei, Taiwan; 52 Faculty of Medicine Siriraj Hospital, Bangkok, Thailand; 53 Faculty of Medicine Chiangmai University, Chiang Mai, Thailand; 54 Faculty of Medicine Vajira Hospital, Navamindradhiraj University, Bangkok, Thailand; 55 Prince of Songkla University, Hat Yai, Thailand; 56 Dubai Coorporation for Ambulance Services, Dubai, United Arab Emirates; 57 Thong Nhat Hospital, Ho Chi Minh City, Vietnam; 58 Viet Tiep Hospital, Haiphong, Vietnam

**Keywords:** Disease prevention, Trauma

## Abstract

Prehospital spinal immobilization is a widely used procedure in the emergency medical service (EMS) system worldwide, while the incidence of patients with spinal injury (SI) is relatively low, and unnecessary prehospital spinal immobilization is associated with patient complications. This study aimed to determine the association between prehospital spine immobilization and favorable functional outcomes at hospital discharge among trauma patients with SI. We conducted a retrospective cohort study using the Pan-Asia Trauma Outcomes Study (PATOS) registry data from January 1, 2016, to November 30, 2018. A total of 759 patients with SI were enrolled from 43,752 trauma patients in the PATOS registry during the study period. The subjects had a median age of 58 years (Q1–Q3, 41–72), and 438 (57.7%) patients had prehospital spine immobilization. Overall, prehospital spinal immobilization was not associated with favorable functional outcomes at discharge in multivariable logistic regression (aOR 1.06; 95% CI 0.62–1.81, p = 0.826). However, in the subgroup of cervical SI, prehospital spinal immobilization was associated with favorable functional outcomes at discharge (aOR 3.14; 95% CI 1.04–9.50; p = 0.043). Therefore, we suggest that paramedics should be more careful when determining the presence of a cervical SI and should apply full spine immobilization if possible.

## Introduction

Patients with spinal injuries (SIs) following trauma are at risk of spinal cord injuries (SCIs) with severe neurological consequences and disability in life, which are estimated in approximately 20% of these cases^1^. However, the incidence of traumatic SCI differs from region to region, with a relatively low incidence of 16 per million in Western Europe to 40 per million in the United States^2^. The incidence of SI in the trauma population also varied from 4.58% in China to all trauma patients^3^ and 9.6% in Europe in patients following major trauma^4^, while some studies reported only 1–2% of all trauma patients^5^.

Despite the relatively low incidence of SI and SCI, prehospital spinal immobilization has been widely adopted worldwide as well as in trauma courses, such as prehospital trauma life support (PHTLS) and advanced trauma life support (ATLS)^6,7^. It aims to minimize further movement of the spine and reduce the risk of secondary injury, but the procedure lacks high-quality evidence of clear benefits in decreasing disability^8,9^ and is associated with complications such as respiratory restriction^10,11^, elevated intracranial pressure^12,13^, pressure ulcers^14^ and changing the results of physical examination^15^ while patients are immobilized. In addition, previous studies also revealed that prehospital immobilization may be associated with higher mortality in patients with penetrating trauma and gun-shot wounds^16–18^.

Due to the growing concerns regarding prehospital spinal immobilization, this study aimed to determine the association between prehospital spinal immobilization and favorable functional outcomes at hospital discharge among adult trauma patients with SI.

## Methods

### Study design and setting

We conducted a retrospective cohort study using a prospectively collected database from the Pan-Asia Trauma Outcomes Study (PATOS), which was a cross-national trauma registry network initiated in 2015 and consists of 33 participating sites from 14 Asian countries, including Australia, Hong Kong, India, Indonesia, Japan, Korea, Malaysia, Philippines, Singapore, Sri Lanka, Taiwan, Thailand, the United Arab Emirates, and Vietnam. Urban areas are covered by all countries^19^. The variables of PATOS include demographics, injury epidemiology, prehospital care, emergency department (ED) and hospital care, injury severity, and clinical outcomes^20^. The PATOS Trauma Database was characterized as an emergency medical service (EMS)-based registry. Participation in the PATOS registry was voluntary. Patient’s data were recorded in the registry if they were sent to the participating hospitals due to trauma, either from the scene or via interhospital transport.

### Study population

We analyzed patients with PATOS from January 1, 2016, to November 30, 2018. Eligible patients were transported by EMS, aged > 16 years, and with SIs. SI was defined as any spinal fracture, dislocation, subluxation, or traumatic disc rupture with or without SCI. The diagnosis was selected using ICD-9 (other paralytic syndromes 344.0–344.9; fracture of vertebral column without mention of SCI 805.0–805.5; fracture of the vertebral column with SCI 806.0–806.6; dislocation of vertebra 839.4; SCI without evidence of spinal bone injury 952.0–952.9) or ICD-10 (fracture of vertebra S12.0–S12.9, S22.0, S32.0, S32.9; dislocation, subluxation of vertebrae, traumatic rupture of intervertebral disc S13.0–S13.1, S23.0–S23.1, S33.0–S33.1; injury of nerves and spinal cord S14.0–S14.9, S24.0–S24.2, S34.0–S34.2) based on the registry data, we excluded patients with pre-existing disability (defined as Glasgow Outcome Score [GOS] < 4 before the injury), traumatic brain injury (TBI) (defined as traumatic cerebral edema, diffuse/focal TBI, epidural hemorrhage, traumatic subdural hemorrhage, traumatic subarachnoid hemorrhage using ICD-9 854.0, 851.0–852.5; ICD-10 S06.1–S06.9), incomplete data on immobilization, Injury Severity Score (ISS), Revised Trauma Score (RTS) at the ED, functional outcome at discharge or prehospital time interval. We excluded patients with TBI because the prevalence of concomitant TBI in patients with an SI can be as high as 32.5%^21^, and we could not differentiate whether the disability resulted from TBI or SI.

### Definition of exposure

The key exposures in our study were prehospital immobilization, which was defined as the neck collar and/or backboard used. We classified the patients using a scoop stretcher as the immobilized group, and previous studies have validated the quality of this immobilization tool compared with that of the spinal board^22,23^. Other recorded immobilization tools such as pelvic binder, femur traction splint, extremity splint and bandaging were classified as non-immobilized group because the primary function of these tools aren’t immobilization of the spine. The basic characteristics of the patients in our study included country, age, sex, mechanism of injury (traffic, fall, others), location of SI (cervical, thoracic, or lumbar spine), and torso injury. Torso injury was defined as injury involving the chest or abdomen, including fractures of the clavicle, rib, pelvis, traumatic pneumothorax, hemothorax, intra-abdominal bleeding, laceration, or contusion of the spleen, liver, or bowel. Data on prehospital management, including spinal immobilization, rescue airway (supraglottic airway or endotracheal tube), the establishment of fluid access either by intravenous line (IV) or by intraosseous line (IO), and scene-to-hospital time (S to H time) were collected. Because one of the major contributing countries (Korea) mostly reported the S to H time rather than transport time, we used S to H time as variables to maximize the valid value. We used the RTS and ISS as indices of trauma severity. RTS is a physiological triage score using the Glasgow Coma Scale (GCS), systolic blood pressure (SBP), and respiratory rate (RR). The RTS formula was as follows: RTS = (GCS score coded × 0.9368) + (SBP coded × 0.7326) + (RR coded × 0.2908)^24^. ISS was calculated by summing the square of the three highest Abbreviated Injury Scale scores for injuries to different body regions^25^. We divided the ISS score into three groups: < 9, minor injury; 9–15 defined as moderate injury; and ≥ 16, severe injury^26,27^. We also collected data from patients who had undergone spine surgery or surgery of other body regions except for the spine. Missing data of the variables were excluded.

### Outcome measurements

The primary outcome was the modified Rankin Scale (mRS) score at discharge. MRS was used to evaluate the functional outcome in patients with stroke at first^28^ and was then widely applied to measure the disability caused by TBI, general trauma and traumatic SI^29–34^. No significant disability to moderate disability (mRS 0–3) was defined as a favorable functional outcome, and moderately severe disability to death (mRS 4–6) was defined as poor functional outcomes^35^. Subgroup analyses were performed in different subgroups of age (< 65 and ≥ 65 years), RTS (< 7 and ≥ 7), ISS (< 9, ≥ 9), and the location of SI (cervical, thoracic, and lumbar regions). We used 65 years old as the cut-off value because it represents the most commonly accepted age to consider a patient as an elderly patient and was used according to the guidelines of the Eastern Association for the Surgery of Trauma^36,37^. A score of 7 as the cut-off point in RTS and 9 as the cut-off point in ISS were used in previous studies for major trauma^27,38,39^.

### Statistical methods

Continuous variables are reported as mean (standard deviation, SD) or median (Q1–Q3), as appropriate. Dichotomous and categorical variables are presented as numbers (percentages). Continuous variables were compared using the Mann–Whitney U test. Categorical and nominal variables were compared using Pearson’s chi-square test or Fisher’s exact test. We examined the association between prehospital spinal immobilization and favorable functional outcomes using univariate logistic regression and multivariable logistic regression. Variables with p < 0.05 on univariable logistic regression and the major variable (prehospital spinal immobilization) were selected for multivariable logistic regression analysis using the forced entry method. For subgroup analyses, we also conducted multiple logistic regression using the forced entry method for all variables. Statistical analysis was performed using SPSS version 25.0 (IBM, Armonk, NY, USA). All tests were two-sided, and a p-value of less than 0.05 was considered statistically significant.

### Ethics approval and consent to participate

The PATOS collaboration was approved by the Institutional Review Board of the National Taiwan University Hospital and Far Eastern Memorial Hospital. This study was conducted in accordance with the Declaration of Helsinki (as revised in 2013) and the study proposal and methods were approved by the PATOS Taipei Meeting. The data were anonymized before being released to the authors in 2019. Institutional Review Board of Far Eastern Memorial Hospital waived the need for Informed consent due to the retrospective nature of this study.

## Results

### Characteristics of study objects

**Figure 1:**
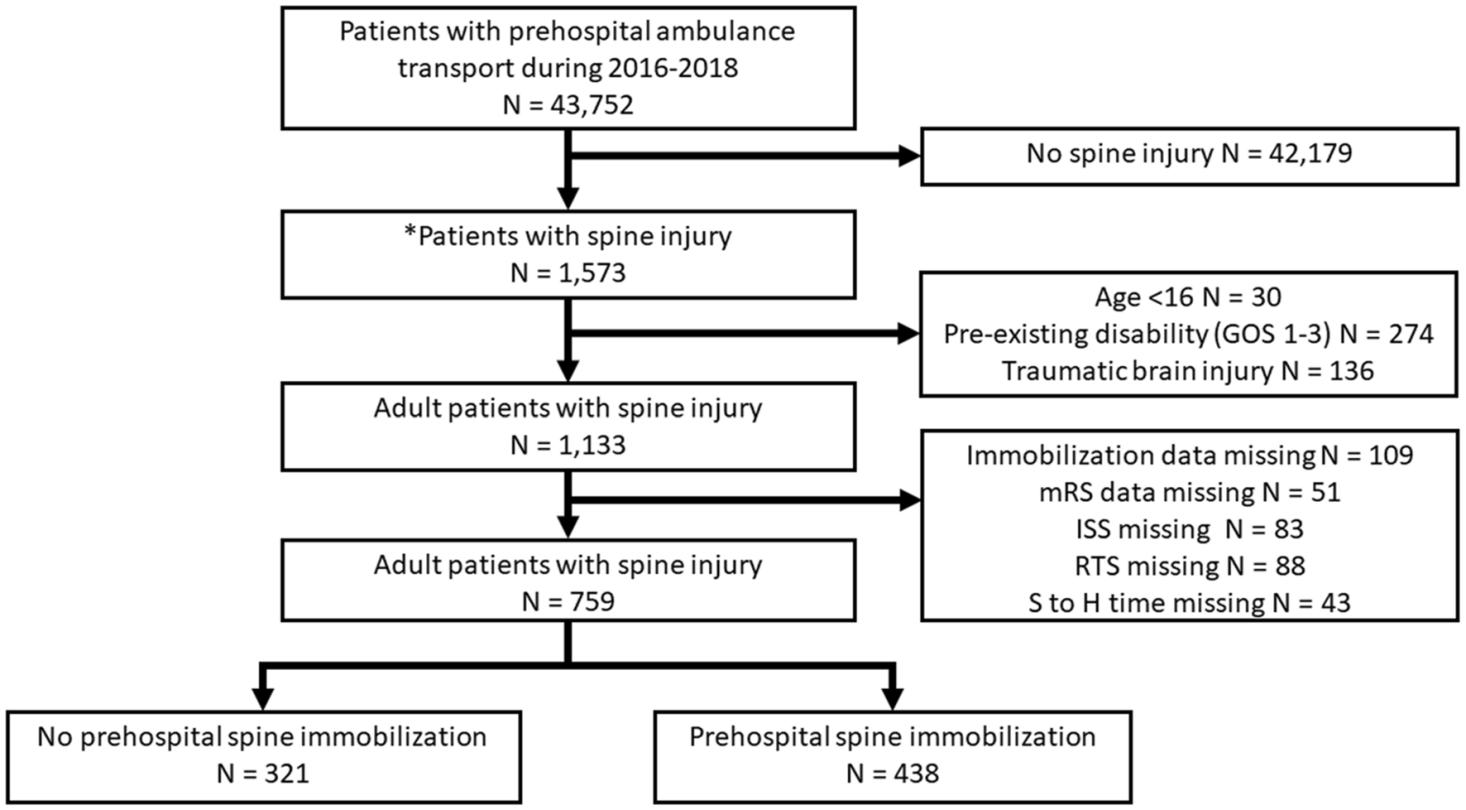
Flow diagram of patients included in our study. *GOS* Glasgow outcome scale, *mRS* modified Rankin Scale, *RTS* revised trauma score, *StoH time* Scene-to-hospital time. *Patients with spinal injury were defined as meeting the diagnosis of ICD-9 or 10 described in the methods.

From January 1, 2016, to November 30, 2018, a total of 1573 cases with SIs were extracted from the PATOS registry of 43,752 EMS-transported trauma patients (3.5% of all cases) using ICD-9 or ICD-10. We further excluded patients aged < 16 years (n = 30), pre-existing disability (GOS 1–3) before the injury (n = 274), TBI (n = 136), and incomplete data (n = 374). The remaining 759 adult patients diagnosed with SI were included in the study, and 438 (57.7%) patients had prehospital spine immobilization. Figure 1 shows a detailed flow diagram of the patients enrolled in the final analysis.

The demographics of the 759 patients with prehospital spinal immobilization are shown in Table 1. The study cohort consisted of patients from four countries, including Korea, Malaysia, Japan, and Vietnam. Korea and Malaysia accounted for 97.1% of the study group. Injury mainly resulted from traffic accidents and falling accidents, and all patients had blunt injuries. The combination of cervical SI and thoracic or lumbar SI was only 3.1% (n = 24). The characteristics of all SI patients without TBI (total number = 1133) are also tabulated in Supplementary Table S1.

**Table 1:** Demographics of immobilized and non-immobilized patients.

		Total N = 759	Immobilized N = 438	Non-immobilized N = 321	P-value
Country N (%)	KR	614 (80.9)	362 (82.6)	252 (78.5)	0.077
	MY	123 (16.2)	74 (16.9)	49 (15.3)	
	OTH^a^	22 (2.9)	2 (0.5)	20 (6.2)	
Age median (Q1–Q3)		58 (41–72)	54 (38–67)	65 (49–76)	< 0.001
Sex N (%)	Female	339 (44.7)	169 (38.6)	170 (53.0)	< 0.001
	Male	420 (55.3)	269 (61.4)	151 (47.0)	
S to H time (median, Q1–Q3)		22 (15–32)	22 (15–33)	22 (15–31)	0.816
Mechanism N (%)	Traffic	318 (41.9)	229 (52.3)	89 (27.7)	< 0.001
	Fall	359 (47.3)	177 (40.4)	182 (56.7)	
	Others^b^	82 (10.8)	32 (7.3)	50 (15.6)	
Prehospital management (no vs. yes)	Fluid (IV, IO)	49 (6.5)	43 (9.8)	6 (1.9)	< 0.001
Location of SI N (%) (no vs. yes)	Cervical SI	201 (26.4)	147 (33.6)	54 (16.8)	< 0.001
	Thoracic SI	197 (26)	113 (25.8)	84 (26.2)	0.909
	Lumbar SI	401 (52.8)	210 (47.9)	191 (59.5)	0.002
	C + T/L	24 (3.1)	22 (5.0)	2 (0.6)	0.001
Torso injury N (%) (no vs. yes)		172 (22.7)	134 (30.6)	38 (11.8)	< 0.001
RTS N (%)	< 7	29 (3.8)	22 (5.0)	7 (2.2)	0.044
	≥ 7	730 (96.2)	416 (95.0)	314 (97.8)	
ISS N (%)	< 9	488 (64.3)	248 (56.6)	240 (74.8)	< 0.001
	9–15	190 (25.0)	137 (31.3)	53 (16.5)	
	≥ 16	81 (10.7)	53 (12.1)	28 (8.7)	
Operation N (%) (no vs. yes)	Spine	61 (8.0)	50 (11.4)	11 (3.4)	< 0.001
	Others^c^	97 (12.8)	73 (16.7)	24 (7.5)	< 0.001
Favorable functional outcome N (%)	Yes	658 (86.7)	371 (84.7)	287 (89.4)	0.059
	No	101 (13.3)	67 (15.3)	34 (10.6)	
Death N (%) (no vs. yes)		7 (0.9)	3 (0.7)	4 (1.2)	0.424

*KR* Korea, *MY* Malaysia, *OTH* others, *S to H time* scene to hospital time, *SI* spinal injury, *RTS* revised trauma score, *ISS* Injury Severity Score.

^a^Others: Japan and Vietnam.

^b^Others: hit by person or object, choking or hanging, drowning, physical overexertion, another mechanism of injury.

^c^Other operations: One patient could have undergone several operations at the same hospital stay.

Compared to the patients in the non-immobilized group, the patients in the immobilized group were younger (54 vs. 65), had a higher percentage of males (61.4% vs. 47.0%), a higher percentage of traffic accidents (52.3% vs. 27.7%), a higher percentage of patients who received prehospital fluid management (9.8% vs. 1.9%), higher percentage of cervical SI (33.6% vs. 16.8%), lower percentage of lumbar SI (47.9% vs. 59.5%), higher percentage of torso injury (30.6% vs. 11.8%), higher percentage of RTS < 7 (5% vs. 2.2%), higher percentage of ISS score 9–15 (31.3% vs. 16.5%) and ≥ 16 (12.1% vs. 8.7%), higher percentage of patients who underwent spine surgery (11.4% vs. 3.4%), and other operations (16.7% vs. 7.5%). We tabulated the GCS scores of the two groups in Supplementary Table S2, and no significant difference was observed.

### Association between prehospital spinal immobilization and favorable functional outcome

For the primary outcome of the association between prehospital spinal immobilization and favorable functional outcome, the demographics and the results of univariable and multivariable logistic regression are shown in Table 2. In univariable logistic regression, prehospital spinal immobilization was not significantly associated with favorable functional outcomes (OR 0.66; 95% CI 0.42–1.02; p = 0.061). Different countries, age, sex, prehospital fluid management, lumbar SI, torso injury, RTS, ISS 9–15, and ISS ≥ 16 was significantly associated with favorable functional outcomes in univariable logistic regression and were selected as adjusting confounding factors in further multivariable logistic regression. In multivariable logistic regression, prehospital spinal immobilization was not associated with favorable functional outcomes (aOR 1.06; 95% CI 0.62–1.81; p = 0.826). Different countries (compared to Korea) (Malaysia aOR 0.37; 95% CI 0.20–0.71; p = 0.002), prehospital fluid management (aOR 0.39; 95% CI 0.19–0.84; p = 0.016), ISS 9–15 (aOR 0.41; 95% CI 0.24–0.72; p = 0.002), ISS ≥ 16 (aOR 0.35; 95% CI 0.17–0.72; p = 0.004), and patients who underwent spine surgery (aOR 0.21; 95% CI 0.11–0.41; p < 0.001) were associated with worse functional outcomes. Only RTS ≥ 7 was associated with favorable functional outcomes (aOR 3.41; 95% CI 1.41–8.25; p = 0.007).

**Table 2:** Demographic, univariable, and multivariable-adjusted logistic regression of favorable and unfavorable functional outcomes in patients.

		Favorable functional outcome N = 658	Unfavorable functional outcome N = 101	Univariable OR (95% CI)	Univariable P-value	Multivariable OR (95% CI)	Multivariable P-value
Country N (%)	KR	551 (83.7)	63 (62.4)	Ref	Ref	Ref	Ref
	MY	89 (13.5)	34 (33.7)	0.30 (0.19–0.48)	< 0.001	0.37 (0.20–0.71)	0.002
	OTH^a^	18 (2.7)	4 (4.0)	0.52 (0.17–1.57)	0.242	0.57 (0.16–1.99)	0.376
Age (median, Q1–Q3)		59 (43–72.3)	52 (34.5–65)	1.02 (1.01–1.03)	0.002	1.00 (0.98–1.01)	0.777
Sex N (%)	Female	308 (46.8)	31 (30.7)	Ref	Ref	Ref	Ref
	Male	350 (53.2)	70 (69.3)	0.50 (0.32–0.79)	0.003	0.76 (0.46–1.27)	0.296
S to H time (median, Q1–Q3)		22 (15.8–31)	23 (14–34.5)	1.00 (0.99–1.01)	0.992		
Mechanism	Traffic	265 (40.3)	53 (52.5)	Ref	Ref		
N (%)	Fall	319 (48.5)	40 (39.6)	1.6 (1.03–2.48)	0.38		
	Others^b^	74 (11.2)	8 (7.9)	1.85 (0.84–4.06)	0.13		
Prehospital management N (%) (no vs. yes)	Fluid (IV, IO)	29 (4.4)	20 (19.8)	5.36 (2.90–9.91)	< 0.001	0.39 (0.19–0.84)	0.016
Location of SI N (%)	Cervical SI	168 (25.5)	33 (32.7)	0.69 (0.41–1.16)	0.158		
(no vs. yes)	Thoracic SI	173 (26.3)	24 (23.8)	1.24 (0.67–2.31)	0.493		
	Lumbar SI	357 (54.3)	44 (43.6)	1.87 (1.17–2.99)	0.009	1.07 (0.67–1.72)	0.772
	C + TLS	21 (3.2)	3 (3.0)	1.08 (0.32–3.68)	0.906		
Torso injury N (%) (no vs. yes)		137 (20.8)	35 (34.7)	0.50 (0.32–0.78)	0.002	1.13 (0.63–2.02)	0.683
Immobilization	No	287 (43.6)	34 (33.7)	Ref	Ref	Ref	Ref
N (%)	Yes	371 (56.4)	67 (67.3)	0.66 (0.42–1.02)	0.061	1.06 (0.62–1.81)	0.826
RTS N (%)	< 7	17 (2.6)	12 (11.9)	Ref	Ref	Ref	Ref
	≥ 7	641 (97.4)	89 (88.1)	5.08 (2.35–11.00)	< 0.001	3.41 (1.41–8.25)	0.007
ISS N (%)	< 9	450 (68.4)	38 (37.6)	Ref	Ref	Ref	Ref
	9–15	150 (22.8)	40 (39.6)	0.32 (0.20–0.51)	< 0.001	0.41 (0.24–0.72)	0.002
	≥ 16	58 (8.8)	23 (22.8)	0.21 (0.12–0.38)	< 0.001	0.35 (0.17–0.72)	0.004
Operation N (%) (no vs. yes)	Spine	39 (5.9)	22 (21.8)	0.23 (0.13–0.40)	< 0.001	0.21 (0.11–0.41)	< 0.001
	Others^c^	75 (11.4)	22 (21.8)	0.46 (0.27–0.79)	< 0.001	0.89 (0.47–1.68)	0.715

*KR* Korea, *MY* Malaysia, *OTH* others, *S to H time* scene to hospital time, *SI* spinal injury, *RTS* revised trauma score, *ISS* Injury Severity Score.

^a^Others: Japan and Vietnam.

^b^Others: hit by person or objects, choking or hanging, drowning, physical overexertion, another mechanism of injury.

^c^Other operations: One patient could have undergone several operations during the same hospital stay.

### Subgroup analysis

**Figure 2:**
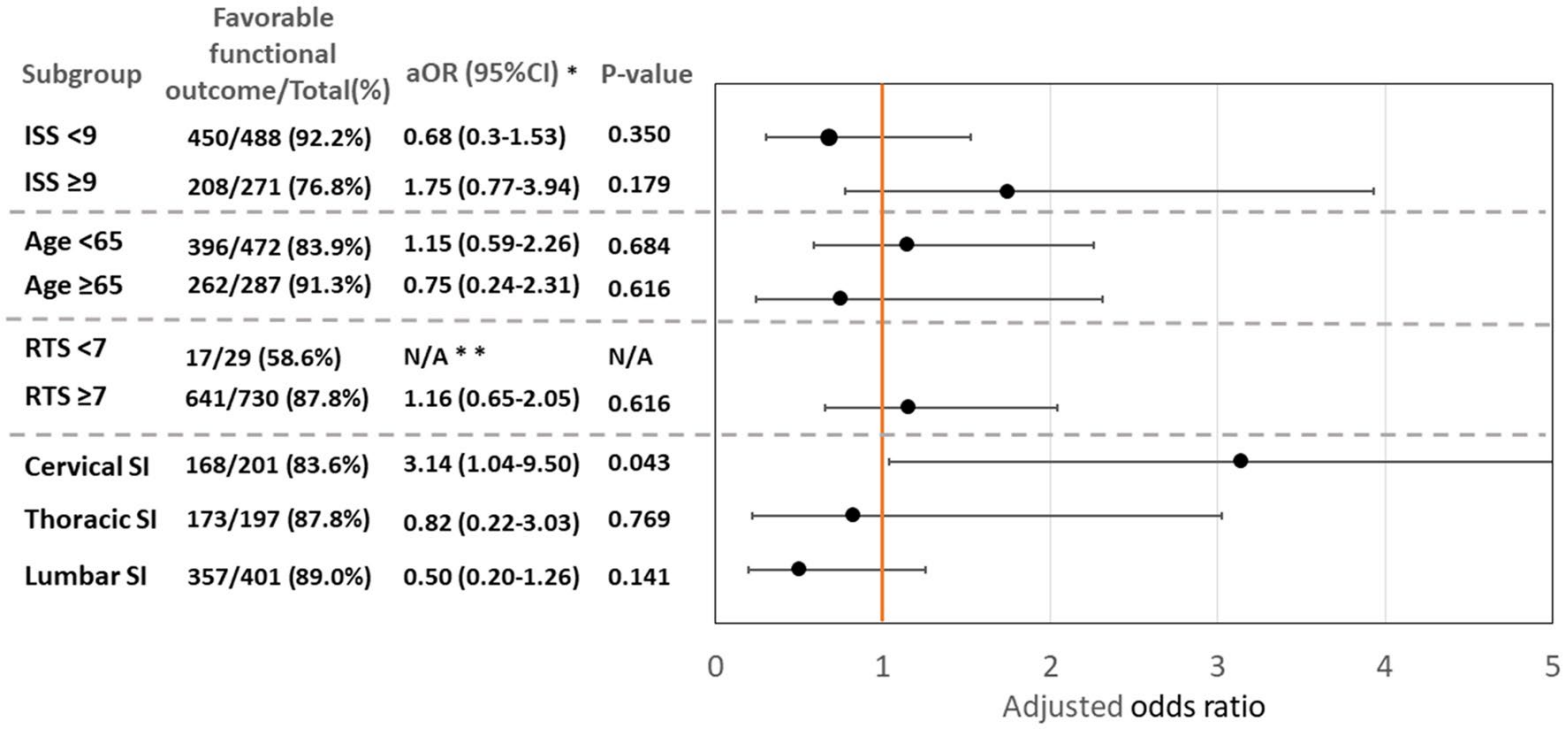
Subgroup analysis for favorable neurologic outcome, ISS < 9, ISS ≥ 9, age ≥ 65, age < 65, adjusted multivariable logistic regression. *aOR* adjusted odds ratio, *SI* spinal injury. *All subgroups except RTS < 7 were adjusted by country, age, sex, scene-to-hospital time, mechanism, prehospital fluid management, cervical spinal injury, thoracic spinal injury, lumbar spinal injury, torso injury, revised trauma score, ISS, received spine operation, and received other operations. **Due to the small subgroup size, multivariable logistic regression was not applicable.

In the subgroup analysis (Fig. 2), we found that prehospital spinal immobilization was associated with favorable functional outcomes in the subgroup of cervical SI (aOR 3.14; 95% CI 1.04–9.50; p = 0.043), but not in the subgroup ISS < 9, ISS ≥ 9, RTS ≥ 7, thoracic SI, and lumbar SI. Unfortunately, multivariable logistic regression could not be performed due to the limited number of patients in subgroup RTS < 7. We further divided the patients with ISS scores of < 9 and ≥ 9 into three groups by injury site (Supplementary Fig. S1). The results revealed that patients with an ISS score of ≥ 9 and cervical SI showed a positive association between immobilization and favorable functional outcomes (aOR 5.50; 95% CI 1.02–29.69; p = 0.048). Moreover, immobilization might cause potential harm in the subgroup with ISS < 9 with lumbar SI.

## Discussion

In this cross-national retrospective cohort study from January 1, 2016, to November 30, 2018, we found that prehospital spinal immobilization was not associated with favorable functional outcomes at discharge in all EMS-transported patients with SI; however, in the subgroup of cervical SI without TBI, the procedure still mattered. There was a significant association between prehospital spinal immobilization and favorable functional outcomes at discharge. Our study has several strengths. First, this was a cross-national study in Asia with different EMS systems. Hence, the results may be applicable to other Asian populations. Second, we excluded preexisting disability before injury and TBI to minimize the effect on functional outcome measured by mRS score at discharge and to focus more on the impact of SI. Although mRS score was not intentionally used to evaluate the traumatic patients with SI, we think that the mRS score provides a more comprehensive assessment of patient’s functional outcome by evaluating the daily activity when compared to the traditionally used American Spinal Injury Association (ASIA) score, which mainly focuses on the evaluation of motor and sensory functions of patients with SI^34^.

The benefit of spinal immobilization came from indirect and low evidence that numerous studies found that delayed diagnosis of SI without immobilization was correlated with worse disability^40–42^. Toscana et al.^42^ conducted a case series of 123 patients with blunt SI and found that 32 (26%) patients had major neurological deterioration between the time of injury and the time of admission. Of these 32 patients, 19 (59%) had no immobilization, suggesting that neurological deterioration resulted from not being immobilized. Meanwhile, Hauswald et al.^43^ retrospectively reviewed patients with traumatic SI from the USA and Malaysia, where the former had prehospital spinal immobilization, but the latter did not have prehospital spinal immobilization in all patients. The results revealed that non-immobilized Malaysian patients had a less neurologic disability (OR 2.03; 95% CI 1.03–3.99; p = 0.04). However, the study was criticized by some points, such as patients who died in the scene or transportation were excluded, no matching of the patient’s injury severity, and relatively small sample size. In our study, we did not exclude patients with traumatic out-of-hospital cardiac arrest, and we performed a subgroup analysis to match the injury severity of patients.

In the subgroup analysis of our study, there was a statistically significant association between prehospital spinal immobilization and favorable functional outcome when limited to patients with cervical SI, especially in patients with an ISS score of ≥ 9. Excluding patients with TBI may be a cause. The prevalence of concomitant TBI in patients with an SI was 32.5% (95% CI 10.8–59.3%) and 40.4% (95% CI 33.0–48.0%) in patients with cervical SI^21^. Severe TBI can cause severe functional disability or death; approximately 38% of patients die^44^ and, as estimated, 43% are discharged with long-term disability^45^. Moreover, spinal immobilization could also increase intracranial cerebral pressure in immobilized patients, which could worsen the situation of TBI^12^. Excluding patients with concomitant TBI decreased the influence of brain injury and helped us focus on the effects of SCI and SI.

The incidence of SI in our study was 3.5% in all EMS-transported trauma patients, which was similar to a previous study from China with an incidence of 4.58% in the trauma population^3^. The percentage of patients with cervical SI and other locations of SI were also similar to those reported in previous studies^3,46^, indicating the generalizability of our findings. The incidence data for Europe and Canada were relatively high, at 9.6% and 23.2%, respectively^4,46^, which may be due to the study population with polytrauma and higher severity in their studies. The leading causes of SI were falls (47.3%) and traffic accidents (41.9%), which is consistent with previous studies^2,47^. The rate of cervical SI combined with thoracic or lumbar SI in our study was 3.1%, which was lower than previous studies (approximately 10%)^4,6^. Patients with lower trauma severity in our study may have been the cause. The rate of non-implementation of spinal immobilization among spine injury patients was relatively high (42%) compared with that reported in Western countries. However, this rate seems compatible with that reported in previous studies in Asia^48,49^. In patients in the immobilized group, statistically significant higher trauma severity (ISS, RTS), higher percentage of combined torso injury, prehospital fluid management, and SI were noted than non-immobilized groups. It is reasonable that severely injured patients would receive more treatment at the scene. Subgroup analysis using ISS and RTS was performed to reduce the impact of injury severity, and the association between favorable functional outcome and prehospital spinal immobilization was not significant in the subgroup with ISS < 9, ISS ≥ 9, and RTS ≥ 7. The scene-to-hospital time was not significantly different between the immobilized and non-immobilized groups, although the immobilized group received more treatment out of the hospital. This might be due to the familiarity of the procedure from paramedics, and only 47% (N = 206) of patients received full spine immobilization in the immobilized group.

The tools used in prehospital immobilization at the scene may also influence the results. Different types of cervical collar and different shapes and angles of the mandible may influence the degree of neck motion^50–52^. The combinations of tools also affect the spine movement^53–55^. Previous cadaver studies have revealed greater neck motion while using cervical collar alone compared with cervical collar with a backboard or vacuum mattress^54,55^. This finding may raise the concern that cervical collar alone may not be sufficient for patients with cervical SI. Hence, full spine immobilization should be performed in these patients. Furthermore, the insertion of a cervical collar may also increase the neck motion in an unstable cervical spine^56,57^, which could also worsen the SI. Spine board with spider straps or vacuum mattress with headblock may also help achieve neck motion restriction compared with a spine board combined with a cervical collar^55^. Hence, it can be another consideration when applying immobilization to patients with cervical SI.

We have requested information regard the prehospital immobilization protocol, tool, technique, and quality from the data stakeholders of the studied countries (Korea, Malaysia, Japan, and Vietnam). Korea and Japan have their own prehospital immobilization protocol, which is similar to the NEXUS criteria. EMTs in Malaysia and Vietnam do provide a written indication for prehospital spinal immobilization, but their training includes the determination of patients who require spinal immobilization.

As for the tools, adjustable Ambu or Laerdal stiff collar and long spinal board were mostly used as immobilization tools in four countries. EMTs in Japan have sometimes used a scoop stretcher (Ferno Scoop 65 EXL) instead of a long spinal board. No vacuum mattress was used in any of the four countries. The use of the same type of immobilization tool minimized the bias of different countries.

Regarding the immobilization technique and quality, the log-roll technique with a patient’s head fixed is the most widely used technique to move patients on the backboard. As for the cervical collar, one paramedic fixes the patient’s head while another EMT inserts an appropriately sized C-collar into the back of the patient's head and fixes the front side under the chin. The quality assessment of the immobilization is also crucial for immobilization, as previous studies revealed that only 11–12% of EMTs applied the backboard and cervical collar without error^58,59^. However, there are no recorded data on the routine quality or technique assessment in our database. We could not determine how the differences among countries would influence our results, and we could not adjust for this unavailable confounder; hence, our analysis was adjusted for the countries included.

### Limitation

There are several limitations to this study. First, this retrospective study had to address the problem of missing data. Some countries did not record some confounding variables, and we had to exclude them from the analysis, which may have caused selection bias. Although we included many variables in the logistic regression analysis, other unknown factors could influence the functional outcomes, such as limited prehospital information from EMS, the quality of each EMS team, bystander management of the patients, the quality and technique of prehospital immobilization at the scene, the neurologic status of patients at the scene, the quality of in-hospital care, and subsequent rehabilitation programs, but these data cannot be included in our analysis because of incomplete data or non-recording of the variables in the registry. Second, The baseline characteristics of the two groups were different; the immobilized group had higher trauma severity (ISS and RTS) and higher percentage of combined torso injury, prehospital fluid management, and SI. This may be a selection bias; we adjusted the differences using univariable, multivariable logistic regression, and subgroup analyses to minimize the influence. Third, the registry data of PATOS were voluntary and could not be considered a representative sample of the included countries. Fourth, the sample size was relatively small. However, considering the relatively low incidence rate of SI and SCI, we believe that these findings are still informative to the knowledge gap.

## Conclusion

Prehospital spinal immobilization was not associated with favorable functional outcomes in traumatic patients with SI; however, subgroup analysis revealed that it may be beneficial for patients with cervical SI without TBI. Based on our findings, we suggest that paramedics should be more judicious when determining the presence of a cervical SI and should apply full spine immobilization if possible. Prospective trials are needed in the future.

## Supplementary Information

Supplementary Figure S1.

Supplementary Information.

## Data Availability

The data that support the findings of this study are available from Pan-Asia Trauma Outcomes Study (PATOS) but restrictions apply to the availability of these data, which were used under license for the current study, and so are not publicly available. Data are however available from the authors upon reasonable request and with permission of PATOS. For more detailed information of PATOS, following website is available, http://lems.re.kr/eng/patos-research/.

## References

[1] Sundstrøm T, Asbjørnsen H, Habiba S, Sunde GA, Wester K (2014). Prehospital use of cervical collars in trauma patients: A critical review. J. Neurotrauma.

[2] Lee BB, Cripps RA, Fitzharris M, Wing PC (2014). The global map for traumatic spinal cord injury epidemiology: Update 2011, global incidence rate. Spinal Cord.

[3] Liu P (2012). Spinal trauma in mainland China from 2001 to 2007: An epidemiological study based on a nationwide database. Spine (Phila Pa 1976).

[4] Hasler RM (2011). Epidemiology and predictors of spinal injury in adult major trauma patients: European cohort study. Eur. Spine J..

[5] Kreinest M, Gliwitzky B, Goller S, Münzberg M (2016). Präklinische Immobilisation der Wirbelsäule. Notfall Rettungsmedizin.

[6] Subcommittee ATLS, American College of Surgeons’ Committee on Trauma, International ATLS Working Group. *Advanced Trauma Life Support*, 10th ed. (2018).

[7] National Association of Emergency Medical Technicians (U.S.), Pre-Hospital Trauma Life Support Committee, American College of Surgeons, Committee on Trauma. *PHTLS: Prehospital Trauma Life Support*. 9th ed. (2018).

[8] Hood N, Considine J (2015). Spinal immobilisaton in pre-hospital and emergency care: A systematic review of the literature. Australas Emerg. Nurs. J..

[9] Purvis TA, Carlin B, Driscoll P (2017). The definite risks and questionable benefits of liberal pre-hospital spinal immobilisation. Am. J. Emerg. Med..

[10] Totten VY, Sugarman DB (1999). Respiratory effects of spinal immobilization. Prehosp. Emerg. Care.

[11] Bauer D, Kowalski R (1988). Effect of spinal immobilization devices on pulmonary function in the healthy, nonsmoking man. Ann. Emerg. Med..

[12] Mobbs RJ, Stoodley MA, Fuller J (2002). Effect of cervical hard collar on intracranial pressure after head injury. ANZ J. Surg..

[13] Núñez-Patiño RA, Rubiano AM, Godoy DA (2020). Impact of cervical collars on intracranial pressure values in traumatic brain injury: A systematic review and meta-analysis of prospective studies. Neurocrit. Care.

[14] Ham WH, Schoonhoven L, Schuurmans MJ, Leenen LP (2016). Pressure ulcers, indentation marks and pain from cervical spine immobilization with extrication collars and headblocks: An observational study. Injury.

[15] March JA, Ausband SC, Brown LH (2002). Changes in physical examination caused by use of spinal immobilization. Prehosp. Emerg. Care.

[16] Brown JB (2009). Prehospital spinal immobilization does not appear to be beneficial and may complicate care following gunshot injury to the torso. J. Trauma.

[17] Haut ER (2010). Spine immobilization in penetrating trauma: More harm than good?. J. Trauma.

[18] Vanderlan WB (2009). Neurologic sequelae of penetrating cervical trauma. Spine (Phila Pa 1976).

[19] Sun KM (2017). Comparison of emergency medical services and trauma care systems among Pan-Asian countries: An international, multicentre, population-based survey. Prehosp. Emerg. Care.

[20] Kong SY (2018). Pan-Asian Trauma Outcomes Study (PATOS): Rationale and methodology of an international and multicenter trauma registry. Prehosp. Emerg. Care.

[21] Pandrich MJ, Demetriades AK (2020). Prevalence of concomitant traumatic cranio-spinal injury: A systematic review and meta-analysis. Neurosurg. Rev..

[22] Del Rossi G, Rechtine GR, Conrad BP, Horodyski M (2010). Are scoop stretchers suitable for use on spine-injured patients?. Am. J. Emerg. Med..

[23] Krell JM (2006). Comparison of the Ferno Scoop Stretcher with the long backboard for spinal immobilization. Prehosp. Emerg. Care.

[24] Champion HR (1989). A revision of the Trauma Score. J. Trauma.

[25] Baker SP, O'Neill B, Haddon W, Long WB (1974). The injury severity score: A method for describing patients with multiple injuries and evaluating emergency care. J. Trauma.

[26] Copes WS (1988). The Injury Severity Score revisited. J. Trauma.

[27] Rozenfeld M (2014). ISS groups: Are we speaking the same language?. Inj. Prev..

[28] van Swieten JC, Koudstaal PJ, Visser MC, Schouten HJ, van Gijn J (1988). Interobserver agreement for the assessment of handicap in stroke patients. Stroke.

[29] Chen C-H (2020). Association between prehospital time and outcome of trauma patients in 4 Asian countries: A cross-national, multicenter cohort study. PLoS Med..

[30] Kohli A (2016). Factors associated with return to work postinjury: Can the modified rankin scale be used to predict return to work?. Am. Surg..

[31] Momenyan S (2017). Predictive validity and inter-rater reliability of the persian version of full outline of unresponsiveness among unconscious patients with traumatic brain injury in an intensive care unit. Neurocrit. Care.

[32] Sadaka F, Patel D, Lakshmanan R (2012). The FOUR score predicts outcome in patients after traumatic brain injury. Neurocrit. Care.

[33] Hsieh SL (2021). Association between the time to definitive care and trauma patient outcomes: Every minute in the golden hour matters. Eur. J. Trauma Emerg. Surg..

[34] Ko JY (2021). The comparison of recovery patterns between ischemic spinal cord injury and traumatic spinal cord injury from acute to chronic phase. J. Spinal Cord Med..

[35] Rangaraju S, Haussen D, Nogueira RG, Nahab F, Frankel M (2017). Comparison of 3-month stroke disability and quality of life across modified rankin scale categories. Interv. Neurol..

[36] Bonne S, Schuerer DJ (2013). Trauma in the older adult: Epidemiology and evolving geriatric trauma principles. Clin. Geriatr. Med..

[37] Jacobs DG (2003). Practice management guidelines for geriatric trauma: The EAST Practice Management Guidelines Work Group. J. Trauma.

[38] Huang Y-T, Huang Y-H, Hsieh C-H, Li C-J, Chiu IM (2019). Comparison of Injury Severity Score, Glasgow Coma Scale, and revised trauma score in predicting the mortality and prolonged ICU stay of traumatic young children: A cross-sectional retrospective study. Emerg. Med. Int..

[39] Jeong JH (2017). The new trauma score (NTS): A modification of the revised trauma score for better trauma mortality prediction. BMC Surg..

[40] Davis JW, Phreaner DL, Hoyt DB, Mackersie RC (1993). The etiology of missed cervical spine injuries. J. Trauma.

[41] Platzer P (2006). Delayed or missed diagnosis of cervical spine injuries. J. Trauma.

[42] Toscano J (1988). Prevention of neurological deterioration before admission to a spinal cord injury unit. Paraplegia.

[43] Hauswald M, Ong G, Tandberg D, Omar Z (1998). Out-of-hospital spinal immobilization: Its effect on neurologic injury. Acad. Emerg. Med..

[44] McIntyre A, Mehta S, Aubut J, Dijkers M, Teasell RW (2013). Mortality among older adults after a traumatic brain injury: A meta-analysis. Brain Inj..

[45] Selassie AW (2008). Incidence of long-term disability following traumatic brain injury hospitalization, United States, 2003. J. Head Trauma Rehabil..

[46] Pirouzmand F (2010). Epidemiological trends of spine and spinal cord injuries in the largest Canadian adult trauma center from 1986 to 2006. J. Neurosurg. Spine.

[47] Ning GZ, Wu Q, Li YL, Feng SQ (2012). Epidemiology of traumatic spinal cord injury in Asia: A systematic review. J. Spinal Cord Med..

[48] Kim YJ, Ahn KO, Song KJ, Shin SD, Suh GJ (2006). Validity of clinical spine clearance criteria for selective pre-hospital spine immobilization. J. Korean Soc. Emerg. Med..

[49] Park J-S, Cho K-J (2020). A study on the current status and the obstacles to prehospital spinal motion restriction performed by 119 paramedics to major trauma patients. Korean J. Emerg. Med. Serv..

[50] Jung MK (2021). Analysis of remaining motion using one innovative upper airway opening cervical collar and two traditional cervical collars. Sci. Rep..

[51] Jung MK, Grützner PA, Schneider NRE, Keil H, Kreinest M (2021). Cervical spine immobilization in patients with a geriatric facial structure: The influence of a geriatric mandible structure on the immobilization quality using a cervical collar. Geriatr. Orthop. Surg. Rehabil..

[52] Rao PJ, Phan K, Mobbs RJ, Wilson D, Ball J (2016). Cervical spine immobilization in the elderly population. J. Spine Surg..

[53] Nolte PC (2021). Analysis of cervical spine immobilization during patient transport in emergency medical services. Eur. J. Trauma Emerg. Surg..

[54] Rahmatalla S, DeShaw J, Stilley J, Denning G, Jennissen C (2019). Comparing the efficacy of methods for immobilizing the cervical spine. Spine (Phila Pa 1976).

[55] Uzun DD (2020). Remaining cervical spine movement under different immobilization techniques. Prehosp. Disaster Med..

[56] Liao S (2018). Motion and dural sac compression in the upper cervical spine during the application of a cervical collar in case of unstable craniocervical junction-A study in two new cadaveric trauma models. PLoS One.

[57] Prasarn ML, Conrad B, Del Rossi G, Horodyski M, Rechtine GR (2012). Motion generated in the unstable cervical spine during the application and removal of cervical immobilization collars. J. Trauma Acute Care Surg..

[58] Kreinest M (2015). Application of cervical collars—An analysis of practical skills of professional emergency medical care providers. PLoS One.

[59] Peery CA, Brice J, White WD (2007). Prehospital spinal immobilization and the backboard quality assessment study. Prehosp. Emerg. Care.

