# Supplementary figures and images for: A multicenter cohort study on the association between prehospital immobilization and functional outcome of patients following spinal injury in Asia
Source: Sci Rep. 2022 Mar 3;12:3492. doi: 10.1038/s41598-022-07481-0 (PMC8894344; doi:10.1038/s41598-022-07481-0)

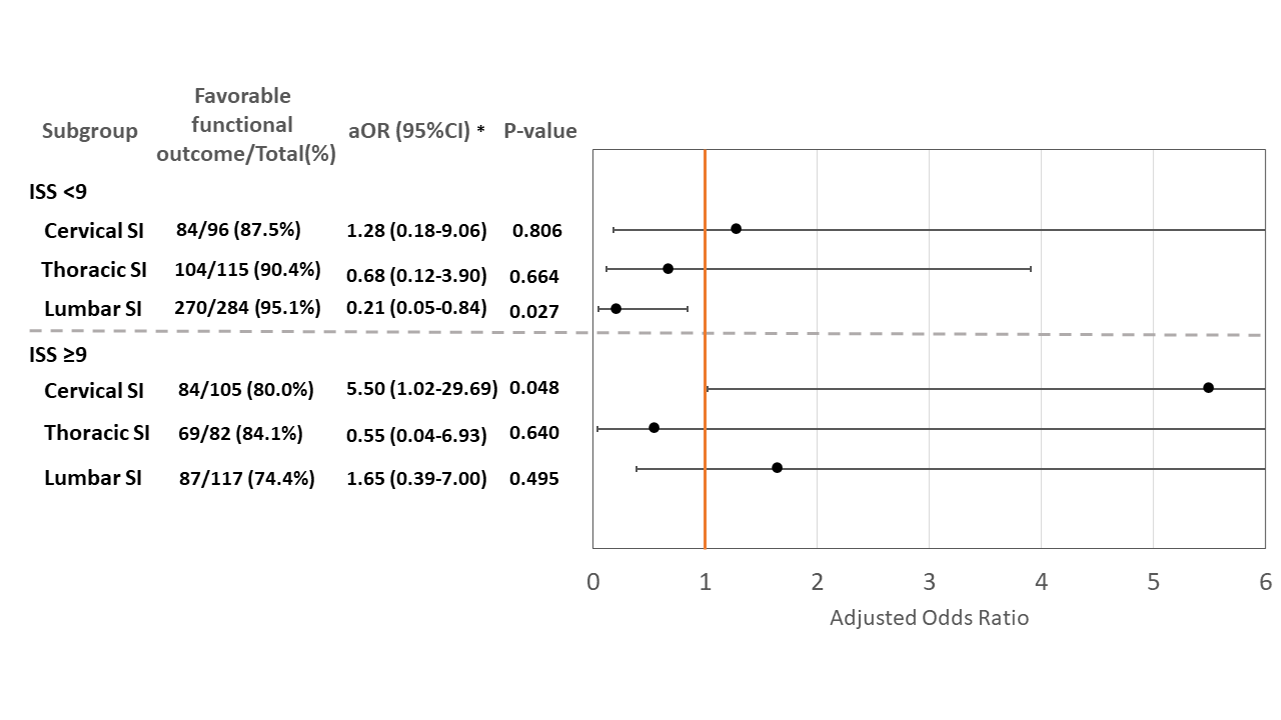

Supplement: Supplementary file 1 — Supplementary Figure S1. [file 41598_2022_7481_MOESM1_ESM.tif]
