# Supplementary material for: A multicenter cohort study on the association between prehospital immobilization and functional outcome of patients following spinal injury in Asia
Source: Sci Rep. 2022 Mar 3;12:3492. doi: 10.1038/s41598-022-07481-0 (PMC8894344; doi:10.1038/s41598-022-07481-0)
Supplement: Supplementary file 2 — Supplementary Information. [file 41598_2022_7481_MOESM2_ESM.docx]

A multicenter cohort study on the association between prehospital immobilization and functional outcome of patients following spinal injury in Asia

Supplementary data

Table S1. Demographics of included patients and patients with any missing of the studied variables or outcomes

|  | | **Total**  **N = 1,133** | **Included**  **N = 759** | **Excluded**  **N = 374** |
| --- | --- | --- | --- | --- |
| **Country** N (%) | KR | 765 (67.5) | 614 (80.9) | 151 (40.4) |
|  | MY | 210 (18.5) | 123 (16.2) | 87 (23.3) |
|  | OTH* | 158 (13.9) | 22 (2.9) | 136 (36.4) |
| **Age** median (Q1-Q3) |  | 56(38−70) | 58(41−72) | 51(34−65) |
| **Sex** N (%) | Female | 481 (42.5) | 339 (44.7) | 142 (38.0) |
|  | Male | 650 (57.4) | 420 (55.3) | 230 (61.5) |
| **S to H time** (median, Q1-Q3) |  | 21(15−30.5) | 22(15−32) | 20(13−28) |
| **Mechanism** | Traffic | 513 (45.3) | 318 (41.9) | 195 (52.3) |
| N (%) | Fall | 515 (45.4) | 359 (47.3) | 156 (41.8) |
|  | Others# | 104 (9.2) | 82 (10.8) | 22 (5.9) |
|  | Missing | 1 (0.1) |  |  |
| **Prehospital management** (no vs. yes) | Fluid (IV, IO) | 83 (7.3) | 49 (6.5) | 34 (12.8) |
|  | Missing | 109(9.6) |  |  |
| **Immobilization** | No  Yes | 437(38.6)  587(51.8) | 321(42.3)  438(57.7) | 116(43.8)  149(56.2) |
|  | Missing | 109(9.6) |  |  |
| **Location of SI** N (%) (no vs. yes) | Cervical SI | 347 (30.6) | 201 (26.5) | 146 (39.0) |
|  | Thoracic SI | 262 (23.1) | 197 (26.0) | 65 (17.4) |
|  | Lumbar SI | 573 (50.6) | 401 (52.8) | 172 (46.0) |
|  | C+T/L | 36 (3.2) | 24 (3.2) | 12 (3.2) |
| **Torso injury** N (%) (no vs. yes) |  | 261 (23.0) | 172 (22.7) | 89 (23.8) |
| **RTS** N (%) | <7 | 54 (4.8) | 29 (3.8) | 25 (10.1) |
|  | ≥7 | 953 (84.1) | 730 (96.2) | 223 (89.9) |
|  | Missing | 126(11.1) |  |  |
| **ISS** N (%) | <9 | 545 (48.1) | 488 (64.3) | 57 (20.5) |
|  | 9−15 | 336 (29.7) | 190 (25.0) | 146 (52.5) |
|  | ≥16 | 156 (13.7) | 81 (10.7) | 75 (27.0) |
|  | Missing | 96(8.5) |  |  |
| **Operation** N (%) (no vs. yes) | Spine | 76 (6.7) | 61 (8.0) | 15 (5.5) |
|  | Missing | 102(9) |  |  |
|  | Others! | 131 (11.5) | 97 (12.8) | 34 (12.5) |
|  | Missing | 102(9) |  |  |
| **Favorable functional outcome** N (%) | Yes | 829 (73.1) | 658 (86.7) | 171 (77.7) |
|  | No | 150 (13.2) | 101 (13.3) | 49 (22.3) |
|  | Missing | 154(13.6) |  |  |
| **Death** N (%) (no vs. yes) |  | 12 (1.0) | 7 (0.9) | 5 (2.3) |
|  | Missing | 154(13.6) |  |  |

KR, Korea; MY, Malaysia; OTH, others; S to H time, scene to hospital time; SI, spinal injury; RTS, revised trauma score; ISS, Injury Severity Score.

*Others: Japan, Vietnam, China, Taiwan, Indonesia

#Others: hit by person or object, choking or hanging, drowning, physical overexertion, another mechanism of injury

!Other operations: One patient could have undergone several operations at the same hospital stay.

Table S2. GCS score at ER in study population*.

| GCS | Total  n=759 | Immobilized  n=438 | Non-immobilized  n=321 | P value |
| --- | --- | --- | --- | --- |
| 13-15 | 734(96.6) | 418(95.4) | 316(98.5) | 0.129 |
| 9-12 | 16(2.1) | 13(3.0) | 3(0.9) |  |
| 6-8 | 7(0.9) | 5(1.1) | 2(0.6) |  |
| 4-5 | 2(0.2) | 2(0.5) | 0(0) |  |
| 3 | 0(0) | 0(0%) | 0(0) |  |

* study population: patients with spinal injury, excluded traumatic brain injury.

**Figure legends**

Figure S1. Association between prehospital spinal immobilization and favorable neurologic outcome in patients with ISS scores of <9 and ≥9 with cervical SI, thoracic SI, and lumbar SI.

aOR, adjusted odds ratio; SI, spinal injury

*All subgroups were adjusted by country, age, sex, scene-to-hospital time, mechanism, prehospital fluid management, cervical spinal injury, thoracic spinal injury, lumbar spinal injury, torso injury, revised trauma score, received spine operation, and received other operations by multivariable logistic regression.
